# The Transcriptomes of the Crucian Carp Complex (*Carassius auratus*) Provide Insights into the Distinction between Unisexual Triploids and Sexual Diploids

**DOI:** 10.3390/ijms15069386

**Published:** 2014-05-27

**Authors:** Chun-Yan Li, Jiong-Tang Li, You-Yi Kuang, Ru Xu, Zi-Xia Zhao, Guang-Yuan Hou, Hong-Wei Liang, Xiao-Wen Sun

**Affiliations:** 1 Heilongjiang River Fisheries Research Institute, Chinese Academy of Fishery Sciences, Harbin 150001, China; E-Mails: xiaoyz0511@163.com (C.-Y.L.); youyikuang@hotmail.com (Y.-Y.K.); 2 College of Fisheries and Life Science, Shanghai Ocean University, Shanghai 201306, China; 3 The Centre for Applied Aquatic Genomics, Chinese Academy of Fishery Sciences, Beijing 100141, China; E-Mails: sdxuru@163.com (R.X.); zhaozx@cafs.ac.cn (Z.-X.Z.); hougy@cafs.ac.cn (G.-Y.H.); 4 Tianjin Fisheries Research Institute, Tianjin 300221, China; 5 College of Life Science and Technology, Dalian Ocean University, Dalian 116023, China; 6 Yangtze River Fisheries Research Institute, Chinese Academy of Fishery Sciences, Wuhan 430223, China; E-Mail: lianghw@yfi.ac.cn

**Keywords:** crucian carp, gibel carp, RNA-seq, genetic diversity, unisexual reproduction

## Abstract

Both sexual reproduction and unisexual reproduction are adaptive strategies for species survival and evolution. Unisexual animals have originated largely by hybridization, which tends to elevate their heterozygosity. However, the extent of genetic diversity resulting from hybridization and the genomic differences that determine the type of reproduction are poorly understood. In *Carassius auratus*, sexual diploids and unisexual triploids coexist. These two forms are similar morphologically but differ markedly in their modes of reproduction. Investigation of their genomic differences will be useful to study genome diversity and the development of reproductive mode. We generated transcriptomes for the unisexual and sexual populations. Genes were identified using homology searches and an *ab initio* method. Estimation of the synonymous substitution rate in the orthologous pairs indicated that the hybridization of gibel carp occurred 2.2 million years ago. Microsatellite genotyping in each individual from the gibel carp population indicated that most gibel carp genes were not tri-allelic. Molecular function and pathway comparisons suggested few gene expansions between them, except for the progesterone-mediated oocyte maturation pathway, which is enriched in gibel carp. Differential expression analysis identified highly expressed genes in gibel carp. The transcriptomes provide information on genetic diversity and genomic differences, which should assist future studies in functional genomics.

## 1. Introduction

Among vertebrates, unisexual individuals have been reported in fish, amphibians, and reptiles [1]. Three modes of unisexual reproduction have been identified, gynogenesis, hybridogenesis, and parthenogenesis [1], but the cellular regulatory mechanisms that maintain unisexual reproduction are still poorly understood. The known unisexual teleost fishes are believed to have arisen by inter-species hybridization of sexual species [2]. Hybridization elevates ploidy and increases genetic diversity of the hybrid at its inception but subsequent genetic drift or diploidization could decrease genomic diversity. Therefore, it is important to explore the genomic diversity of hybrid vertebrates. Comparative genomic analysis between unisexual animals and their closely related sexual species will provide clues to the mechanisms of regulation of unisexual reproduction and allows estimation of the genomic diversity in the hybrid species.

*Carassius auratus* complex is characterized by the coexistence of sexual diploids and unisexual triploids [3]. The diploids and the triploids are quite similar morphologically but differ markedly in their modes of reproduction. The diploid form has 100 chromosomes and reproduces sexually. In the sexual reproduction, the sperm nuclei are able to transform into male pronuclei and fuse with the eggs. According to current taxonomy, the triploid individuals belong to the subspecies *Carassius auratus gibelio*, also named gibel carp, silver crucian carp, or prussian carp [4]. Gibel carp is believed to have originated by the ancient hybridization of a diploid female crucian carp gamete and a male common carp genome gamete [5]. It possesses 156–162 chromosomes [6,7] and has dual reproduction modes of unisexual gynogenesis and sexual reproduction [8,9]. Gynogenesis by heterogeneous spermatozoa activation is the dominant mode of reproduction and produces all female triploid offspring. In gynogenesis, gibel carp egg development is activated by sperm of other fish, but the incorporated sperm nucleus is kept in condensation and fails to form a male pronucleus [10]. Therefore, the heterologous sperm does not contribute genetically to the offspring. Sexual reproduction, the minor mode, generates sexual triploid progenies. It is estimated that a male ratio of about 20% in gibel carp population are produced from the sexual reproduction [11]. The difference of sexual reproduction and unisexual gynogenesis is likely related to some unknown regulatory mechanisms. The co-existence of the sexual form and the unisexual form makes this complex a promising model in which to study the mechanisms underlying their distinct reproductive modes and genetic diversity following hybridization.

*C. auratus* complex exhibits an additional round of genome duplication compared with other teleosts and this feature has been used to study the consequences of genome duplication [12]. In addition, fish in the *C. auratus* complex are hypoxia tolerant but the mechanisms are not fully understood [13]. These special genetic and phenotypic characteristics suggest that the *C. auratus* complex may be a suitable model to study genome duplication and physiological adaptation. Considering the promising applications of *C. auratus* in reproductive biology, genome duplication, and adaptive evolution, construction of the genomic resources of the complex will facilitate applications of this system in a number of fields of study.

In this research, we performed RNA sequencing for gibel carps and for diploid crucian carps to determine functional differences between them, and differentially expressed genes. We also investigated tri-allelic polymorphism in gibel carp. These studies provide hints on the genetic diversity within unisexual fish and the regulatory mechanisms concerned with unisexual reproduction. The transcriptomes of the crucian carp complex provide a representative resource for further functional and comparative analyses.

## 2. Results and Discussion

### 2.1. Transcriptome Sequencing and Assembly

**Figure 1:**
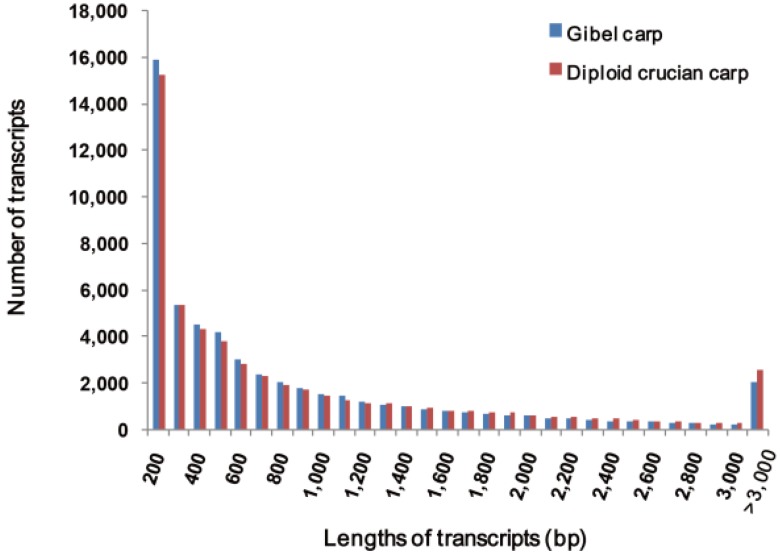
The length distributions of gibel carp transcripts and diploid crucian carp transcripts.

The diploid and triploid individuals are quite similar morphologically and are difficult to differentiate by their appearance. The ploidy of the sequenced populations was determined by flow cytometry (Figure S1). The mean DNA content of gibel carps is 480, which is 1.5 times that of the diploid crucian carps (320). The ratio is consistent with their different ploidy types. Transcriptome sequencing yielded 11,669,953 and 12,135,538 pairs of reads for gibel carps and diploid crucian carps, respectively. Raw RNA-sequencing reads have been deposited at the NCBI Sequence Read Archive (SRA) under accession numbers SRR922167 and SRR924100. After filtering out the low-quality bases and *de novo* assembly, 65,476 and 67,297 transcripts were obtained in gibel carp and crucian carp, respectively. Based on sequence similarity, transcripts were clustered into genes and the longest transcript in each gene was selected as representative. Finally, we obtained 54,459 non-redundant transcripts (an N50 length of 1463 bp) in gibel carp and 53,839 non-redundant transcripts (an N50 length of 1672 bp) in diploid crucian carp. The length distributions of all sequences in the two fish are shown in Figure 1.

One goal of this study was to construct a representative transcriptome resource for the crucian carp complex. We applied saturation analysis to ascertain whether sequencing coverage was sufficient to draw a comprehensive picture of the transcriptome for the crucian carp complex. For each fish, rarefied libraries were constructed by randomly sampling from 10% to 100% of the transcriptome data. Then we produced new assemblies at each of the defined levels to illustrate possible differences in gene discovery rates. The curve for each species was already saturated (Figure S2), indicating that a large part of the genes were detected and that our study provided a comprehensive transcriptome resource for the crucian carp complex. Recently, Liao *et al*., generated transcriptomes for four tissues of diploid crucian carp and identified differentially expressed genes among four tissues [14]. However, the transcriptome was collected from only four tissues, leading to short transcripts (N50: 547 bp) and a large number of transcripts (127,711 unigenes). Compared with their result, our data provides a more representative collection of diploid crucian carp genes. In addition, we provide the most comprehensive transcriptome resource for gibel carp to date.

### 2.2. Sequence Annotation and Validation

To identify the protein-coding genes, we first carried out homolog searches. Homolog searches against fish Ensembl proteins, fish Ensembl transcripts, the NCBI non-redundant protein (nr) database, and UTRdb revealed 46,630 putative protein-coding genes in gibel carp and 44,402 in diploid crucian carp (Table 1). The *ab initio* prediction identified an additional 433 and 1260 coding genes in these two fish, respectively. Because these transcripts had no hits to known proteins, it is possible that they are species-specific protein-coding genes. The proportions of protein-coding transcripts among all transcripts here were 85.6% in gibel carp and 82.5% in diploid crucian carp. These values are higher than those recorded in other transcriptome studies in non-model organisms [15,16,17].

**Table 1:** Summary of the annotation of transcripts of gibel carp and diploid crucian carp.

	Database	Gibel Carp	Diploid Crucian Carp
Homolog search	Fish Ensembl proteins	32,030	31,620
	Fish Ensembl transcripts	13,513	10,956
	NCBI “nr” protein database	235	56
	UTRdb	852	1770
	NONCODE database	336	491
	NCBI “nt” transcript database	4004	1386
*ab initio* prediction	BESTORF	433	1260
Unknown		3056	6300
Total		54,459	53,839

In gibel carp, we also found 4340 transcripts either homologous to known non-coding genes in the NONCODE database or aligned to NCBI “nt” transcripts, indicating that they may be non-coding RNAs (ncRNAs). A total of 1877 transcripts in diploid crucian carp were putative ncRNAs. The remaining unknown 3056 gibel carp transcripts and 6300 diploid crucian carp transcripts had neither protein-coding potential nor known homologs, indicating that they were probably transcribed from intergenic regions of the gibel carp and diploid crucian carp genomes.

To evaluate the accuracy of our assemblies, 12 pairs of paralogs in gibel carp and diploid crucian carp were randomly selected for RT-PCR and specific primers were designed for the selected transcripts. All of the selected transcripts could be amplified (Figure S3), indicating that they were actually expressed and correctly assembled.

### 2.3. Genome Speciation Event Deduced from Orthologous Pairs

**Figure 2:**
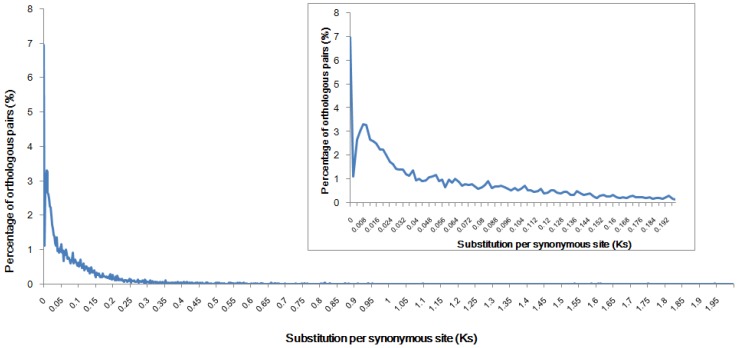
*Ks* distribution for identification of the speciation event. Data were grouped into bins of 0.002 *Ks* units for graphing. The *Ks* distribution of the orthologous pairs is plotted as the blue line and shows a distinct secondary peak, indicating the time of divergence of these two species.

The speciation time between diploid crucian carp and gibel carp has not been reported previously. A secondary peak in the distribution of orthologous *Ks* values indicates a speciation event [18]. We estimated the genome speciation time based on the *Ks* distribution of orthologous pairs between the two species. We identified 18,974 orthologous pairs between diploid crucian carp and gibel carp using the reciprocal best blast hit approach. The *Ks* distribution of these orthologous pairs showed a distinct secondary peak at 0.008 (with a mode at *Ks* = 0.006 to 0.01) (Figure 2).

For analysis of speciation time, we used the rate of 3.51 × 10^−9^ substitutions/synonymous site per year obtained for fourfold degenerate sites of 47 mammalian genes [19]. The rate was estimated using the method of Pamilo and Bianchi [20], assuming that the divergence time between the human and rodent lineages was 80 million years ago. With the constant clock-like rate, the speciation between diploid crucian carp and gibel carp was estimated to have occurred 2.2 million years ago (with a range of 1.7–2.8 million years). It is believed that the triploid gynogen originated from an ancient hybridization event with crucian carp the maternal ancestor and common carp (*Cyprinus carpio*) the paternal ancestor [5]. Therefore, this is possibly one of the most recent inter-species hybridizations among vertebrates.

### 2.4. Low Tri-Allelic Polymorphism and Heterozygosity in Gibel Carp

Hybridization could elevate the genetic diversity of the unisexual species at its inception [1]. However, subsequent ploidy restoration could lead to greatly reduced or completely absent heterozygosity [21,22]. In addition, it is well known that sexual reproduction increases the genetic diversity of offspring while gynogenesis does not. Therefore, it is interesting to compare the genomic diversity between gibel carp and diploid crucian carp. We identified 628,711 polymorphic sites in 21,615 diploid crucian carp transcripts and 509,699 sites in 22,463 gibel carp transcripts. The average diversity level of gibel carp was 22.7 polymorphic sites per transcript, lower than the one of diploid crucian carp (29.1 sites per transcript).

Given the tri-ploidy in gibel carp, theoretically tri-allelic polymorphism might be observed in many genomic regions. However, the frequency of tri-allelic polymorphism in gibel carp is unknown. To investigate the level of tri-allelic polymorphism, we plotted the allele number per polymorphic site. Among 509,699 polymorphic sites in gibel carp, only 7681 belonged to tri-allelic polymorphism while the remaining loci were di-allelic.

The di-allelic polymorphism in the triploid population could come from the following genotypes in individuals, including AAB, BBA, BBB, or AAA. Because di-allelic polymorphism was prevalent in the genome, mono-allelic genotypes and di-allelic genotypes widely existed in most polymorphic loci while the tri-allelic genotypes (ABC) would distribute in only few loci. To validate our hypothesis, we further investigated the proportion of tri-allelic genotypes by microsatellite genotyping in each individual. A total of 3953 microsatellites were identified in 3258 gibel carp transcripts and 4153 microsatellites in 3382 diploid crucian carp transcripts. In each of the two species, the top microsatellites were di-nucleotide motifs (54.2% and 53.7%, Table S1). Eighteen conserved microsatellite loci were genotyped in the gibel carp population. This analysis showed that most loci were still homozygous or di-allelic heterozygous while the loci of tri-allelic heterozygosity accounted for only 22% (Table S2).

A previous study using microsatellite genotyping in 94 gibel carp individuals also found that most loci were homozygous or diploid heterozygous [23]. One explanation of the low tri-allelic polymorphism is that the two inter-breeding species were genetically very similar. The extant triploid represents the fusion of a diploid female crucian carp gamete (AB) and a male common carp genome gamete (C). The various genotypes of triploids can be classified on the basis of their genome constitution, as ABC (if the three alleles are heterozygous to one another), AAC (if A and B are identical), and AAA (if all three alleles are identical). Crucian carp and common carp both belong to the family Cyprinidae and because of their great similarity both AAC and AAA types would be prevalent in the triploid genome. An alternative explanation is that genetic drift following the hybridization event pushed most loci to fixation. In the ancient triploid, each locus might initially have had three alleles. If a reasonable length of time elapses before the species becomes diploidized, genetic drift could cause some loci to become fixed for alleles that originated from one parent, whereas other loci might retain three alleles from the two parents. A third possible explanation is that diploidization followed inter-species hybridization. Diploidization is assumed to occur through the accumulation of DNA sequence mutations and/or deletions between sister chromosomes [24], leading to the loss of alleles. The diploidized loci then exhibit homozygosity or di-allelic heterozygosity. Summarizing, whatever the explanation for the low frequency of tri-allelic polymorphisms, investigations into tri-allelic polymorphisms will open the door to understanding the whole-genome ploidy level of other polyploids that originated by hybridization.

Microsatellites and SNPs identified within these two species will be useful for future molecular selection and breeding. Because most transcripts in the two fish were protein-coding, the identified SNPs were mainly located in protein-coding genes. The SNPs in the coding regions could change protein sequences and functions. In addition, SNPs in the UTRs might affect the regulation of miRNAs to target genes [25]. Therefore, the SNPs mined here may help to advance functional studies of SNPs and the identification of phenotype-associated SNPs.

### 2.5. Few Gene Expansions in Gibel Carp

As described above, only a few genomic loci in gibel carp showed tri-allelic heterozygosity, reflecting the evolution of ploidy after hybridization. To investigate gene expansions in gibel carp resulting from inter-species hybridization, we compared molecular functions and pathways between the two forms. Such comparisons may also indicate possible mechanisms underlying the phenotypic differences. Using homologous assignment, we assigned 4041 GO terms to 50.1% of the gibel carp transcripts (27,310 of 54,459). A similar percentage was assigned in diploid crucian carp (50%, 4066 terms to 26,957 transcripts). These two fish shared 3891 GO terms, indicating that most of their molecular functions were common to the two fish. We then used WEGO to find significantly enriched GO terms in gibel carp using diploid crucian carp transcripts as the background. In the molecular function category, only four GO terms, including binding and transferase activity, were significantly overrepresented while four terms, mainly transporter activities, were underrepresented in gibel carp (Table 2).

KEGG pathway analysis mapped 7132 gibel carp transcripts to 162 zebrafish KEGG pathways. A total of 6966 diploid crucian carp transcripts were mapped to 162 zebrafish pathways and used as a background to compare pathway differences. The statistically enriched pathways are shown in Table 3. Only three pathways were enriched in gibel carp and four pathways in diploid crucian carp, consistent with the observation of few GO differences between them. The consistency of these two independent analyses indicates that few genes are expanded in gibel carp.

**Table 2:** The enriched molecular functions in gibel carp and diploid crucian carp (*p* value < 0.05).

Species	GO Term	GO ID	Percentage of Transcripts (%) *	*p* Value
Gibel carp	nucleic acid binding	GO:0003676	13.9:13.2	0.009
	transferase activity	GO:0016740	10.4:9.8	0.012
	transferase activity, transferring phosphorus-containing groups	GO:0016772	6.6:6.2	0.028
	DNA binding	GO:0003677	7.6:7.1	0.033
Diploid crucian carp	transporter activity	GO:0005215	4.1:4.7	0.000
	transmembrane transporter activity	GO:0022857	3.2:3.9	0.000
	passive transmembrane transporter activity	GO:0022803	1.0:1.4	0.000
	substrate-specific transmembrane transporter activity	GO:0022891	2.7:3.3	0.000
	substrate-specific transporter activity	GO:0022892	4.1:4.7	0.001

* Percentage of transcripts: the first number is the proportion of gibel carp transcripts in this molecular function and the second number is the percentage of diploid crucian carp transcripts.

**Table 3:** The enriched pathways in gibel carp and diploid crucian carp identified by KOBAS (*p* value < 0.05).

Species	KEGG Pathway	Percentage of Gibel Carp Transcripts (%)	Percentage of Diploid Crucian Carp Transcripts (%)	*p* Value
Gibel carp	Ubiquitin mediated proteolysis	1.70	1.38	0.013
	Progesterone-mediated oocyte maturation	1.13	0.89	0.018
	Fanconi anemia pathway	0.61	0.46	0.038
Diploid crucian carp	Neuroactive ligand-receptor interaction	1.07	1.53	0.000
	Cell adhesion molecules	0.91	1.18	0.010
	Ribosome	0.55	0.74	0.024
	Tight junction	1.51	1.76	0.045

Gene expansion or loss may lead to some enriched functions or pathways in one species. As expected, most molecular functions and pathways were common to these two fish and only a small number were enriched in each species. These differences in molecular functions and pathways might be responsible for the unisexual gynogenesis of gibel carp. Interestingly, the gibel carp transcripts were enriched in the progesterone-mediated oocyte maturation pathway. This pathway is reported to increase the maturation promoting factor (MPF) [26], which might lead to the tripolar spindle formation and the later modification of microtubule dynamics [27]. The enriched GO terms in this fish, concerned with transferase activity and the transferring phosphorus-containing groups, are much related involved in the enriched progesterone-mediated oocyte maturation pathway [28]. The ubiquitin-mediated proteolysis pathway and the Fanconi Anemia pathway were also significantly enriched in gibel carp. The MPF consists of *cdc2* and *cyclin B* [29]. The degradation of cyclins is essential to regulate the transition from mitosis to the next cell cycle and is regulated by the ubiquitin-mediated proteolytic pathway [30]. The Fanconi Anemia pathway participates in DNA repair and restores chromosomal integrity [31]. These two pathways might also be genetic mechanisms responsible for unisexual gynogenesis in gibel carp.

### 2.6. Differentially Expressed Orthologous Genes in Pooled Tissues and Gonads

**Figure 3:**
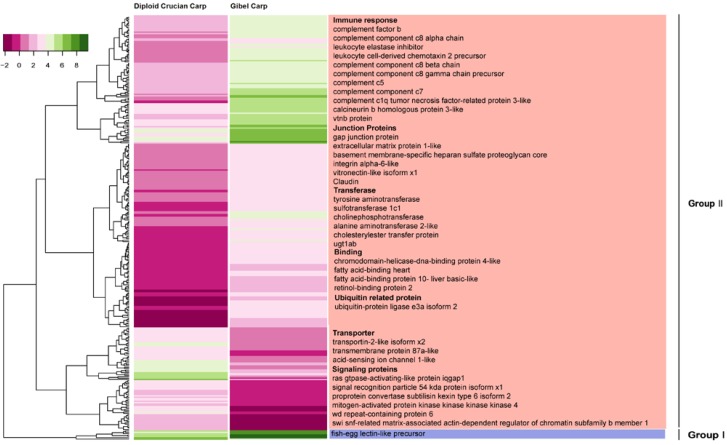
Expression profiling of differentially expressed orthologous genes in the two species. Hierarchical clustering shows that these genes can be classified into two groups (Group I and Group II). The group II could be further divided into two types.

Gene expansion analysis can provide hints on the possible mechanisms leading to phenotypic differences. Differential gene expression analysis may also help us investigate these mechanisms. We identified 314 differentially expressed genes (DEGs) in the pooled tissues between diploid crucian carp and gibel carp, which accounted for only 1.7% of orthologous pairs. That is, most of the orthologous genes had similar expression levels in these two forms. Hierarchical clustering on the basis of expression patterns indicated that the DEGs were classified into two major groups (Figure 3). In the first group, the expression of orthologous genes was high in both fish but was higher in gibel carp than in diploid crucian carp. There were seven genes in this group. Of particular interest was the fish-egg lectin-like precursor, which is reported to have a function in embryonic development [32]. How this precursor functions in the specialized mode of reproduction of gibel carp needs further investigation.

In the second group, the orthologs were highly expressed in one form but had low expression in the other. This group was further divided into two types: (1) highly expressed in diploid crucian carp; (2) highly expressed in gibel carp. Of 307 genes in the second group, 76 belonged to the first type while most of the DEGs (75%, 231 out of 307) were attributed into the second type. The genes of the first type were transporter-associated genes or signaling protein-associated genes. In contrast, the highly expressed genes in gibel carp were concerned with the immune-response, cell junctions, transferases, and binding functions.

**Figure 4:**
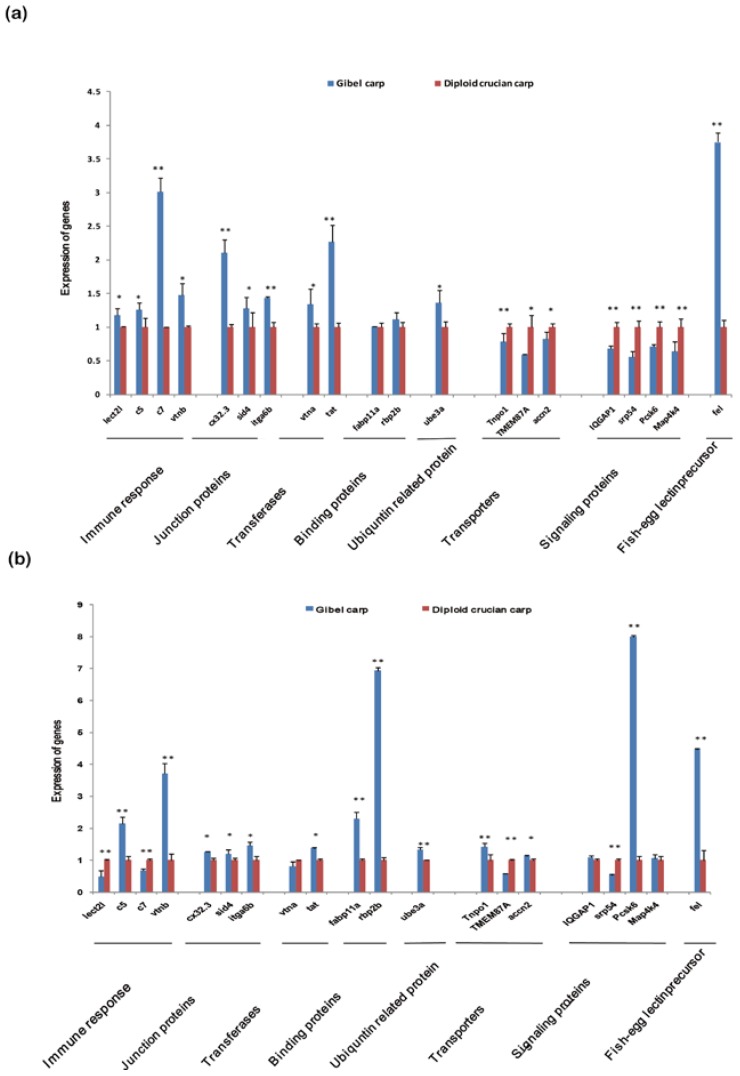
Differentially expressed genes validated by RT-qPCR. (**a**) The expression levels of 20 DEGs were quantified by RT-qPCR in pooled tissues of diploid crucian carps (red) and gibel carps (blue). The *x*-axis shows genes in different categories; the *y*-axis displays the gene expression level (Student’s *t-*test; * *p* < 0.05, ** *p* < 0.01). The expression patterns of most DEGs by RT-qPCR were similar to those in the RNA-Seq analysis; (**b**) The expression levels of 20 DEGs were quantified by RT-qPCR in gonads of diploid crucian carp (red) and gibel carps (blue) (Student’s *t-*test; * *p* < 0.05, ** *p* < 0.01).

To validate our RNA-Seq results, 20 genes with statistically significant differential expression were selected for quantitative reverse transcription-polymerase chain reaction (RT-qPCR) analysis in the two populations. These genes participated in functions associated with the immune-response, cell junctions, transferases, transporter activities, molecular binding, and signaling. Overall, except for two genes without significant differences, the expression patterns of 18 genes by RT-qPCR were significantly different and similar to those indicated by the RNA-Seq analysis (Figure 4a). Thus, the similar patterns suggested by RNA-Seq analysis and RT-qPCR validated the genome-wide expression profiles in pooled tissues of the two subspecies.

The differential expression analysis with the RNA-Seq and RT-qPCR suggests that these molecular functions might be involved in the phenotype differences. The most important phenotypic differences relate to their modes of reproduction. Therefore, we further investigated the expression patterns of these 20 genes in the gonads with RT-qPCR. Of 17 genes with significant difference, the patterns of 12 (71%) in the gonads were consistent with those in the pooled tissues (Figure 4b). Interestingly, some immune-response genes, including *c5* and *vtnb*, were up-regulated in gibel carp gonads compared with diploid crucian carp. It was reported that the complement 1q (*C1q*) family of proteins function as both immunological mediators and in vertebrate oogenesis and oocyte maturation [33]. Hence, these immune-response proteins in gibel carp may play roles in the special mode of unisexual reproduction. These data also demonstrated up-regulation of other genes in gibel carp gonads. In particular, the fish-egg lectin-like precursor was markedly up-regulated more than 4.5 times in gibel carp gonads. This up-regulation is consistent with our observation in the pooled tissues. Fish-egg lectin was reported to be expressed differently in gibel carp and diploid crucian carp and up-regulated in gibel carp [34]. Our comparative data are consistent with this study.

Transcriptome comparison provided another layer of possible mechanisms for phenotypic differences resulting from differences in gene expression. Although the comparison demonstrated that most orthologs had similar expression levels, our analysis identified some differentially expressed genes. Taken together, these data provide important information on the distinction between unisexual triploids and sexual diploids.

## 3. Experimental Section

### 3.1. Sample Collection and Transcriptome Sequencing

We collected two populations of mature female gibel carp and female diploid crucian carp (each population, *N* = 10, 100–150 g, non-infectious) from Yangtze River Fisheries Research Institute, Wuhan, China. Use of the samples for all experiments was approved by the Ethics Committee of the institution. The sex of each fish was determined by examination of the gonads. The ploidy types of these fish were confirmed based on their DNA contents using Cell Lab Quanta SC flow cytometer (Beckman Coulter, Brea, CA, USA). Red blood cells were collected from the caudal vein of each individual with syringes containing sodium heparin. The blood samples were resuspended in Nuclear Isolation Media (NIM)-DAPI staining solution (NPE Systems, Pembroke Pines, FL, USA) for 10 min. The DNA content of each individual was compared with that of chicken. The DNA of each sample was isolated using the QIAamp DNA Blood Mini Kit (Qiagen, Hilden, Germany). DNA concentrations were measured with NanoVue Plus Spectrophotometer (GE Healthcare, Little Chalfont, UK) and the integrity was confirmed by analysis on a 1% agarose gel.

In each fish, tissue samples were excised from the brain, muscle, liver, skin, kidney, gill, intestine, gonad, spleen, and heart. Total RNA was extracted from each of the ten tissues using the Trizol Kit (Invitrogen, Carlsbad, CA, USA) and followed by DNase I (Invitrogen, Carlsbad, CA, USA) treatment according to the manufacturer’s protocol. For each individual, equal quantities (1 µg) of the RNA from the ten tissues were pooled. Finally, the pooled RNA from ten individuals of the same population was checked with Bioanalyzer 2100 (Agilent Technologies, Santa Clara, CA, USA) for the integrity. The RNA integrity number (RIN) values for both fish were 7.8, indicating that the quality satisfied the following commercial Illumina sequencing and RT-qPCR [35].

The library construction from purifying mRNA to enriching DNA fragments was performed with the Illumina TruSeq RNA Sample Prep Kit (Illumina, San Diego, CA, USA). Briefly, for each fish, polyA mRNA was extracted from 10 µg RNA using poly-T oligo attached magnetic beads. During the elution of the polyA RNA, the RNA was fragmented and primed with random hexamers using the fragment and prime mix (Illumina, San Diego, CA, USA). Following the Prep Kit, the purified RNA was fragmented under the conditions: 94 °C for 8 min followed by a final hold at 4 °C. The fragmentation resulted in libraries with sizes ranging from 300 to 400 bp. The cleaved RNA fragments primed with random hexamers were reversely transcribed into first strand cDNA with Superscript II reverse transcriptase (Invitrogen, Carlsbad, CA, USA). The process was performed under the following conditions: incubation at 25 °C for 10 min followed by 15 min at 42 °C; then 70 °C for 15 min; final hold at 4 °C. After the second strand cDNA was synthesized and a single “A” nucleotide was added to the 3' end of the blunt ds cDNA fragment, the adapters with a single “T” nucleotide on the 3' end were ligated to the fragments. Finally, those DNA fragments with adapters on both ends were enriched using PCR. The amplification was performed under the conditions: initial denaturation at 98 °C for 30 s; then 15 cycles at 98 °C for 10 s, at 60 °C for 30 s, and then at 72 °C for 30 s; final extension at 72 °C for 5 min. The libraries were then sequenced on an Illumina HiSeq 2000 platform (Illumina, Inc., San Diego, CA, USA) with read lengths of 2 × 100 nt.

### 3.2. Transcriptome Assembly, Annotation, and Validation

For each individual, the raw transcriptome reads were processed using SolexaQA [36] to filter low-quality reads. The high-quality reads were assembled using Trinity [37]. The Trinity contigs were further assembled using CAP3 [38]. Because the similarity between two crucian carp paralogs was as high as 96% [39], to avoid misassembly of paralogs into one gene, we set the overlap percent identity cutoff as 97. That is, if two transcripts had an overlapping region with an identity over 97%, they were considered from the same gene and should be assembled further. The output contigs were subjected to SSPACE, a stand-alone scaffolder of pre-assembled contigs using paired-read data [40], for scaffolding. To avoid the identification of redundant genes as a result of alternative splicing, we followed the strategy of Wang *et al.* [17], where all-against-all BLASTN (National Center for Biotechnology Information, Bethesda, MD, USA) searches were performed using SSPACE transcripts. If the alignment of two transcripts had 100% identity over 100 bp, then these two transcripts were considered as spliced variants and the longest transcript was selected to represent this gene.

To ascertain whether sequencing depth was sufficient to draw a comprehensive picture of the transcriptome for the crucian carp complex, we constructed different libraries by randomly sampling from 10% to 100% of the transcriptome data. The reads from different libraries were assembled using Trinity and CAP3. Then, the assembled transcript numbers were plotted for different read numbers. A curve was drawn to indicate if sequencing effort was deep enough.

To identify protein-coding genes, we used a combination of homolog searches and an *ab initio* prediction method. First, we searched homologous fish proteins (zebrafish, fugu, tetraodon, medaka, and stickleback) for our assembled transcripts using BLASTX (National Center for Biotechnology Information, Bethesda, MD, USA) with an e-value of 10^−5^. The e-value cutoff was used in many homology detection strategies [41,42]. The fish proteins were obtained from the Ensembl database [43]. The transcripts without BLASTX hits were then searched against fish transcripts (zebrafish, fugu, tetraodon, medaka, and stickleback) using BLASTN. To identify as many homologous genes as possible, we increased the e-value cutoff to 10^−2^. The transcripts were downloaded from the Ensembl database [43] and the UCSC genome database [44]. Transcripts that had no match to either of these databases were aligned against the NCBI “nr” protein database using BLASTX. The remaining unmatched transcripts were further aligned against UTRdb [45] using BLASTN. The hit transcripts were considered to be the untranslated regions (UTRs) of protein-coding genes. For the transcripts without hits to UTRdb, we carried out a BLASTN search against the NONCODE database [46] and the NCBI “nt” nucleotide database. Second, for the transcripts without homology to the above databases, we predicted their putative open reading frames (ORFs) using BESTORF (http://linux1.softberry.com/berry.phtml?topic=bestorf&group=programs&subgroup=gfind). Because species-specific protein-coding genes exist, these genes could not be identified using homolog searches. Because short putative ORFs could be predicted by chance within noncoding RNAs (ncRNAs), a minimum ORF cutoff is usually applied to reduce the likelihood of falsely categorizing ncRNAs as mRNAs [47]. To distinguish mRNAs from transcriptomes, we adopted a cutoff of 150 nt (50 amino acids) [48]. That is, if BESTORF predicted an intact ORF over 50 amino acids, this transcript was considered as a protein-coding gene.

To annotate the functions of the genes, we assigned Gene Ontology information [49] of homologous fish proteins to gibel carp genes and crucian carp genes. To study the pathways that the genes may participate in, we ran KOBAS software [50] to map transcripts to zebrafish pathways in the Kyoto Encyclopedia of Genes and Genomes (KEGG) [51].

To validate the assembled transcripts, the pooled RNAs were used for cDNA synthesis. We randomly selected 12 pairs of paralogs and designed specific primers for them (Table S3). PCR reactions were conducted in a 15 μL volume containing 300 ng cDNA, 10 μM primers, 10× universal PCR buffer, and 0.5 U of *Taq* polymerase (Fermentas, Burlington, ON, Canada). PCR was performed under the following conditions: initial denaturation at 95 °C for 5 min; then 35 cycles at 94 °C for 30 s, at a primer-specific annealing temperature for 30 s, and then at 72 °C for 45 s; final extension at 72 °C for 10 min. PCR products were then checked on a 1.5% agarose gel.

### 3.3. Genome Speciation Event Deduced from the Ks Distribution of Orthologous Pairs

Since there are no available annotated protein sequences or complete genome sequences for these two fish, the proteome-based ortholog detection strategies, such as Inparanoid [52] and OrthoMCL [41], are not suitable for our analysis. To identify reliable orthologs between gibel carp and diploid crucian carp, we adopted the BLAST-based Reciprocal Best Hit (RBH) method by our previous strategy [17] and Blanc *et al.* [18]. The sequences from the two species were aligned using the reciprocal BLAST (BLASTN) hit method. If each of two aligned sequences was the best hit of the other, and if they were aligned over 300 bp, they were defined as orthologs. Chen *et al.* concluded that the BLAST-based RBH method had a low false positive rate of 8% [53]. The RBH method has been widely applied into identifying orthologs in those species of which proteins were not annotated [18,53].

The approach used to estimate the *Ks* of orthologous pairs was also adapted from previous strategy [18,54]. Briefly, the gibel carp transcripts were aligned with their orthologous diploid crucian carp transcripts with TBLASTX. The longest alignment was selected for analysis. In each pair, the corresponding aligned sequence was extracted and translated with the *getorf* program in the EMBOSS package [55]. Then, the translated amino acid sequences were aligned using Clustalw [56]. The corresponding codon alignments were generated using PAL2NAL [57]. Finally, we calculated the *Ks* of each orthologous pair using a maximum likelihood method in the CODEML program (runmode-2) of the PAML package [58].

### 3.4. The Analysis of Tri-Allelic Polymorphism and Heterozygosity in Gibel Carp

To compare the diversity level between two populations and study the proportion of tri-allelic polymorphism in gibel carp, we estimated the SNP allele number per polymorphic locus. For each species, we aligned the sequencing reads to the representative transcripts with the CLC Genomics Workbench (http://www.clcbio.com/products/clc-genomics-workbench/). The general alignment parameters were set to the default values except that non-specific matches were ignored. We adjusted the minimum read coverage to 5 [15].

The prevalent di-allelic polymorphism in the population indicated that the tri-allelic genotypes (ABC) would exist in only few loci in genome. Thus, we examined the proportion of the tri-allelic heterozygosity by genotyping microsatellite loci in each individual of the sequencing population. Microsatellites were identified using Msatfinder (http://www.genomics.ceh.ac.uk/msatfinder/). The repeat thresholds for di-, tri-, tetra-, penta-, and hexa-nucleotide motifs were set as 8, 5, 5, 5, and 5 respectively. Only microsatellite sequences with flanking sequences longer than 50 bp on both sides were collected. Eighteen microsatellites (Table S4, Supplementary Information) were genotyped in individuals following the pipeline of Zhang *et al.* [59].

### 3.5. Gene Expansions in Special Molecular Functions and Pathways

As a consequence of inter-species hybridization, the proportion of genes in a protein family might be higher in gibel carp than in diploid crucian carp, leading to species-specific gene expansion of particular protein-families, and enriched molecular functions. This preliminary comparative study of gene expansion in molecular functions could provide clues concerning the functions that might underlie the phenotypic differences between these two fish, particularly in their reproductive modes. To study the distinct molecular functions and pathways within the different phenotypes, we identified significantly enriched GO terms in gibel carp transcripts using diploid crucian carp genes as the background using WEGO [60]. Terms with *p* value <0.05 were considered to be enriched in gibel carp. To study the enriched pathways in gibel carp, we compared the proportion of gibel carp transcripts in each KEGG pathway against the proportion of diploid crucian carp genes in the same pathway using KOBAS, assuming a hypergeometric distribution. If the proportion of gibel carp transcripts in one KEGG pathway was significantly higher than that of diploid crucian carp transcripts (*p* value < 0.05), this KEGG pathway was considered statistically enriched in gibel carp.

### 3.6. Analysis and Validation of Differentially Expressed Orthologous Genes

Differential gene expression might also explain the phenotypic differences. Sequencing reads were mapped to the assembled reference transcriptomes of the two species using Bowtie [61]. RSEM (RNA-Seq by Expectation Maximization), an accurate method of transcript quantification from RNA-Seq data with or without a reference genome [62], was used to estimate the gene abundance. The expressions of orthologous genes were joined and then normalized with edgeR (Empirical analysis of digital gene expression data in R) [63]. After pair-wise comparison, the differentially expressed genes (DEGs) were obtained with stringent cutoffs: FDR (false discovery rate)-corrected *p* value cutoff of 0.001 and minimum fold change of 4. The FDR correction is designed to control the expected proportion of incorrectly rejected null hypotheses and used in multiple-hypothesis testing to reduce Type-1 errors [64].

To categorize the DEGs according to their expression patterns, a heat map chart was constructed by transforming the normalized data to a log2 scale for visualization. Hierarchical clustering on the basis of expression was performed using the “gplots” package of the R program. The DEGs were further clustered into different groups based on their functional annotations.

We selected twenty DEGs and used RT-qPCR to validate their expression levels in the pooled tissues and in the gonads of triploid gibel carp and diploid crucian carp. The pooled RNA samples were used for RT-qPCR. We followed the Minimum Information for Publication of Quantitative Real-Time PCR Experiments (MIQE) Guidelines [65] for quality assessment. Purity of the four RNA samples was checked by measuring the *A260/A280* ratio with a NanoVue Plus Spectrophotometer (GE Healthcare, Little Chalfont, UK). These samples all demonstrated high purities with ratios over 2.0 (Table S5). RNA integrities were assessed with Bioanalyzer 2100 (Agilent Technologies, Santa Clara, CA, USA). For each sample, clearly visible 28S and 18S rRNA peaks demonstrated the high integrity (Figure S4). The RIN values for theses samples were 7.8 or higher (Table S6), also indicating the high integrity [66,67]. Then, cDNA was synthesized using about 3 µg of total RNA with the RevertAid™ H Minus First Strand cDNA Synthesis Kit (Fermentas, Burlington, ON, Canada). The beta-actin gene was used as the reference gene because of its validated stable expression in different tissues and conditions of crucian carp [68,69]. Since β-actin has been widely applied in gene expression as an internal control gene in crucian carp [70,71], we consider that the expression of β-actin is stable and could be used as an efficient and single reference gene in our study [72,73]. To determine whether inhibition was occurring during PCR, β-actin was amplified in different dilution series of cDNA. The amplification plots in these samples were showed in Figure S5. The significant linear correlation between quantification cycle values (*C*q) and DNA concentrations demonstrated the absence of inhibition during PCR. The primers of the analyzed genes were shown in Table S7. RT-qPCR was performed on an ABI PRISM 7500 Real-time Detection System (Applied Biosystems, Foster City, CA, USA). The amplification was performed in a total volume of 15 μL, containing 7.5 μL 2× SYBR Green Realtime PCR Master Mix (Toyobo, Osaka, Japan), 1 μL cDNA (100 ng/μL), and 0.3 μL of 10 μM of each gene-specific primer. The PCR cycle was 50 °C for 2 min, 95 °C for 10 min, 40 cycles of 95 °C for 15 s, and 60 °C for 1 min. All reactions were set up in triplicate. The amplification efficiencies of the twenty DEGs and the reference gene were 1, calculated with 7500 Real-Time PCR System version 2.0 software (Applied Biosystems, Foster City, CA, USA). The expression of each DEG was normalized to that of beta-actin. The comparative *C*q method (2^−ΔΔ*C*q^ method) was used to analyze the expression of the target genes. After normalization, the levels of the genes of gibel carp are stated relative to the orthologous genes of diploid crucian carp. Data are expressed as means ± SE. Statistical differences between the two groups were determined by the Student’s *t*-test.

## 4. Conclusions

In this study, we performed transcriptome sequencing for two forms of *C. auratus*, one reproducing sexually and the other unisexually. The assembled transcriptomes and their annotations permitted functional characterization and comparative analysis. The analysis enabled us to investigate: (1) the extent of tri-allelic polymorphism in gibel carp; and (2) the putative regulatory mechanisms for their different modes of reproduction. The transcriptome sequences, annotations, microsatellites, and SNPs obtained in this study will be valuable resources for basic research, including investigations of reproductive biology, genome duplication, and physiological adaptation.

## Supplementary Files

Supplementary File 1 Supplementary Information (PDF, 449 KB)
