# Supplementary material for: The Transcriptomes of the Crucian Carp Complex (Carassius auratus) Provide Insights into the Distinction between Unisexual Triploids and Sexual Diploids
Source: Int J Mol Sci. 2014 May 27;15(6):9386–406. doi: 10.3390/ijms15069386 (PMC4100101; doi:10.3390/ijms15069386)
Supplement: Supplementary File 1 — Supplementary Information (PDF, 449 KB) [file ijms-15-09386-s001.pdf]

Supplementary Information

Figure S1. DNA contents of gibel carps and diploid crucian carps measured by flow cytometry using chicken (G1) as the reference. The *x*-axis shows DNA content, and the *y*-axis is cell number. (**A**) exhibits DNA contents for chicken (G1), diploid crucian carp (G2) and gibel carp (G3); (**B**–**K**) show DNA contents of ten diploid crucian carps (G2) using chicken DNA content (G1) as the reference; (**L**–**U**) display DNA contents of ten gibel carps (G2) and chicken (G1). The mean DNA content of gibel carp population is 1.5 times that of the diploid crucian carps.

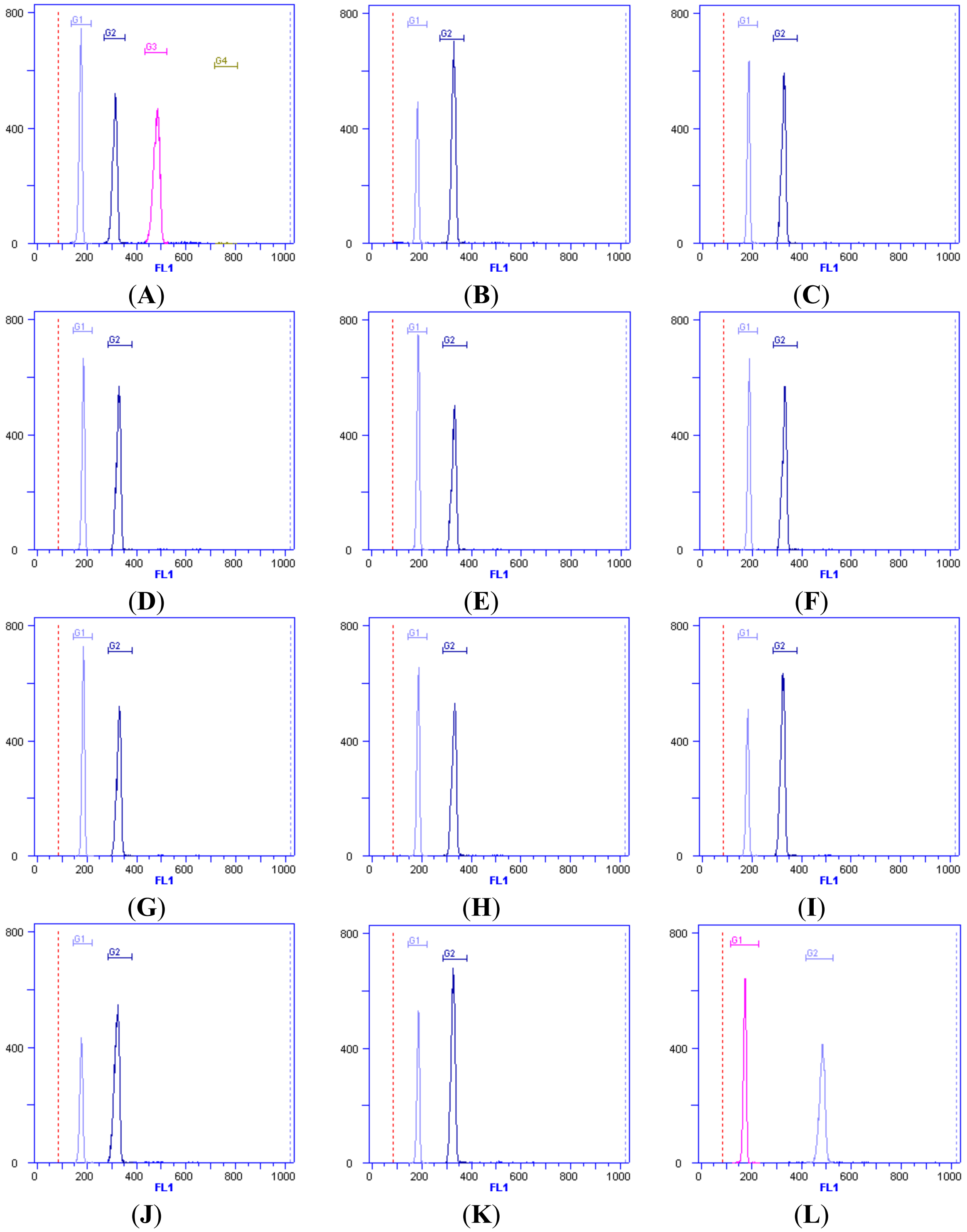

Figure S1. *Cont.*

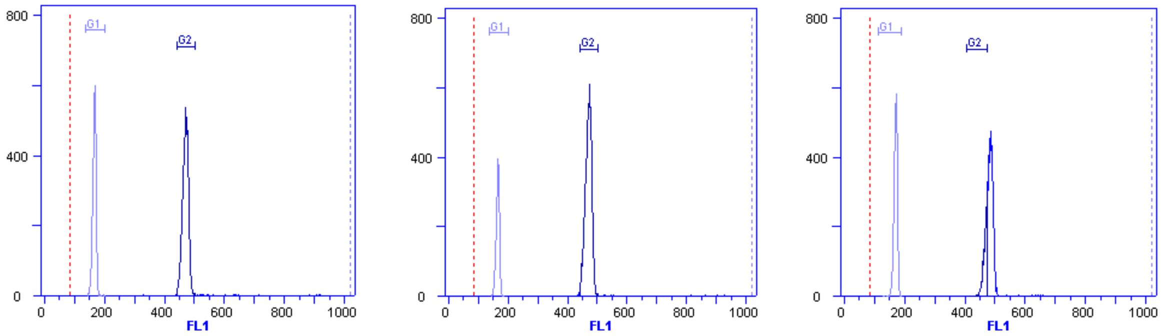

(M) (N) (O)

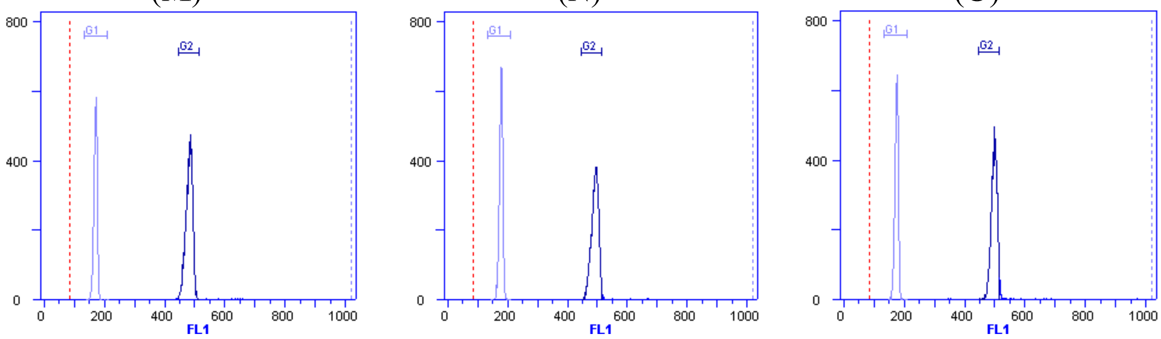

(P) (Q) (R)

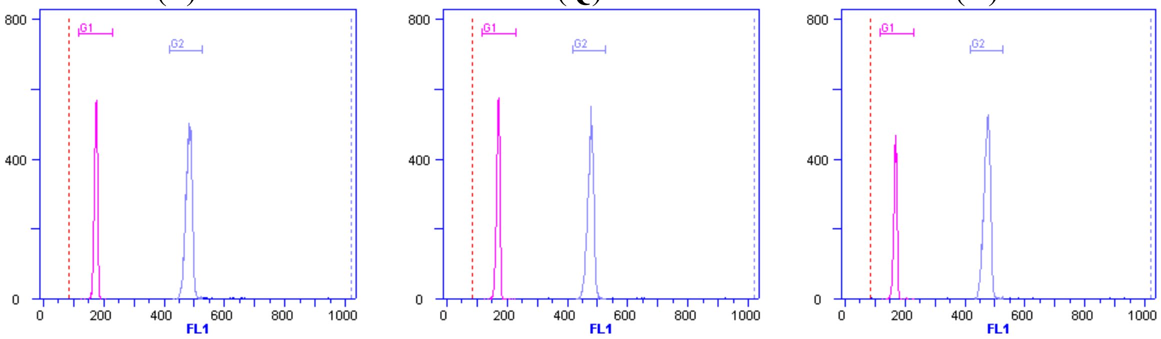

(S) (T) (U)

Figure S2. Saturation analysis for transcriptome coverage. Saturation curves for two species representing expected gene coverage rates. Curves were constructed for two fish.

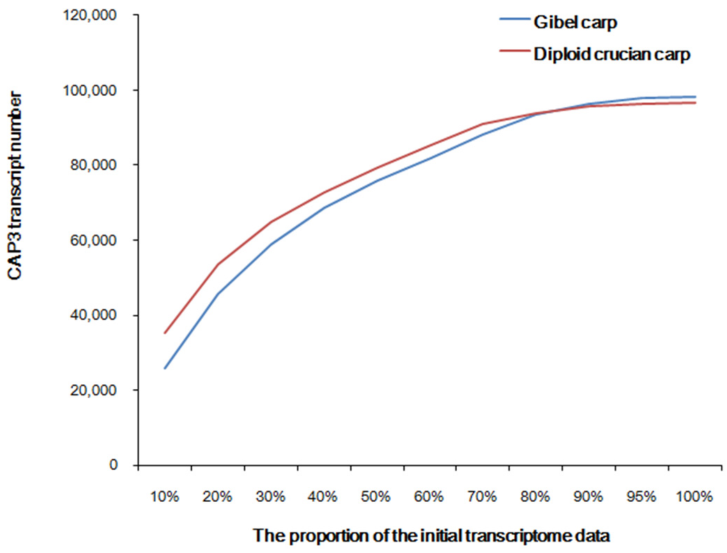

Figure S3. (**A**) 1–12: Gibel carp conserved genes; 13: common carp β-actin; M: DL2000 DNA marker; (**B**) 1–12: Diploid crucian carp conserved genes; 13: common carp β-actin; M: DL2000 DNA marker.

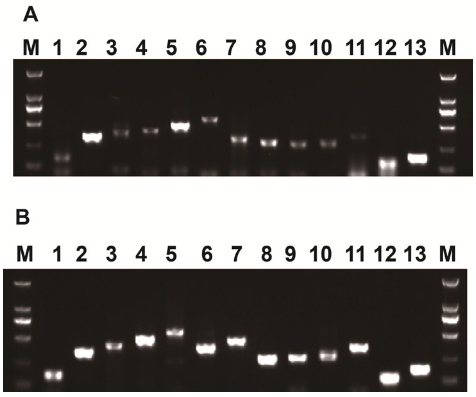

Figure S4. Gel electrophoresis with Bioanalyzer 2100. L: ladder; 1: pooled tissues of triploid gibel carp; 2: pooled tissues of diploid crucian carp; 3: gonad of triploid gibel carp; 4: gonad of diploid crucian carp.

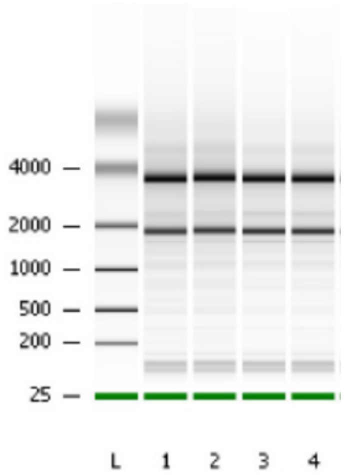

Figure S5. Amplification plots of beta-actin in the 1:10 dilution test. (**A**) 1:10 serial dilution series of cDNA from the pooled tissues of triploid gibel carp; (**B**) 1:10 serial dilution series of cDNA from the pooled tissues of diploid gibel carp; (**C**) 1:10 serial dilution series of cDNA from the gonad of triploid gibel carp; (**D**) 1:10 serial dilution series of cDNA from the gonad of diploid gibel carp.

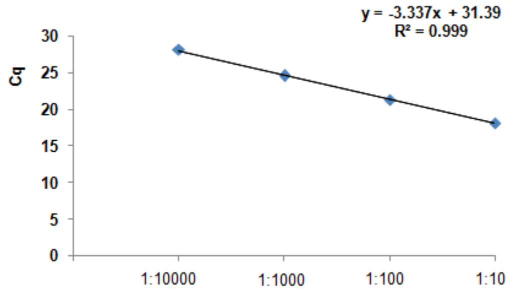

(**A**)

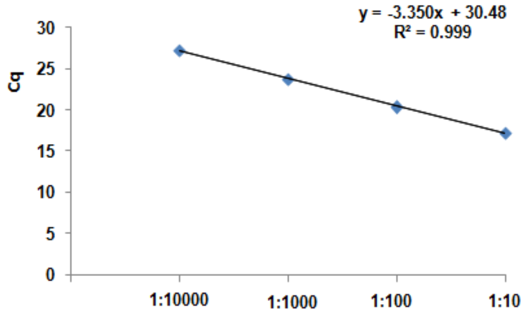

(**B**)

Figure S5. *Cont.*

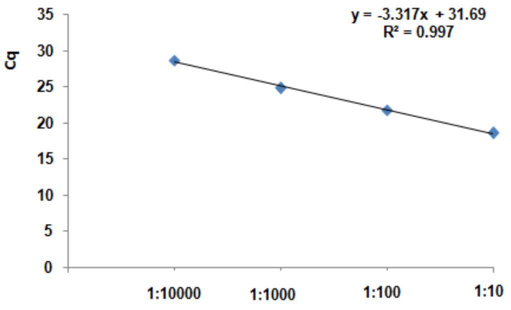

(**C**)

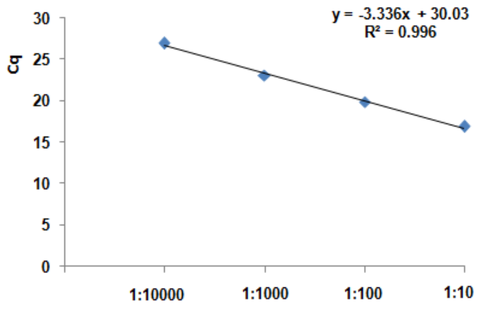

(**D**)

Table S1. Summary of microsatellites identified from the transcriptomes.

Type	Number	
	Gibel Carp	Diploid Crucian Carp
Di-nucleotide repeats	2141	2229
Tri-nucleotide repeats	1641	1754
Tetra-nucleotide repeats	155	158
Penta-nucleotide repeats	8	3
Hexa-nucleotide repeats	8	9
Total number of identified SSRs	3953	4153
Number of transcripts containing SSRs	3258	3382

Table S2. The level of tri-allelic heterozygosity estimated by microsatellite genotyping in individuals.

Loci	*Gibel Carp*	Sample									
	Transcript	1	2	3	4	5	6	7	8	9	10
1 [$]	000000377	2 [%]	2	1 [※]	2	2	2	2	2	3	2
2	000002772	2	2	3 [#]	2	2	2	2	1	3	2
3	000003185	1	1	3	1	1	2	2	2	1	2
4	000003195	N [&]	1	2	3	3	2	3	2	2	3
5	000006001	2	2	3	1	3	2	2	2	3	3
6	000003781	1	1	1	1	1	1	1	1	1	1
7	000003916	2	2	1	2	1	1	1	2	2	1
8	000003932	1	1	1	1	1	1	1	1	1	1
9	000001091	2	3	3	1	3	3	3	3	2	N
10	000001234	1	2	2	2	2	1	2	2	2	1
11	000001414	1	2	2	3	3	3	2	3	3	2
12	000004045	N	N	N	1	1	N	N	N	1	N
13	000004497	2	2	1	2	2	2	2	2	2	3
14	000005660	N	2	3	3	3	2	3	3	N	N
15	000006377	2	2	2	3	N	2	2	1	2	1
16	000006392	2	1	2	2	2	2	2	3	2	2
17	000007745	2	1	N	1	1	1	1	2	3	2
18	000033229	3	3	1	2	2	N	2	3	N	N

[※] 1 represents homozygous sites; [%] 2 represents di-allelic polymorphism; [#] 3 represents tri-allelic polymorphic sites; [&] N represents no amplification; [$] the information of the eighteen loci was showed in Table S4 (Supplementary Information).

Table S3. Specific primers to amplify the assembled transcripts.

Loci	*Gibel Carp* Transcript	Diploid *Crucian Carp* Transcript	Forward Primer	Reverse Primer
1	000006377	fedi000000971	AGATGCTTGAACTTGAGAACCCTG	AGGCAAGGCACTATACAACG
2	000014179	fedi000005970	ATTTCAGAGCGAGGGAGC	CCTGGAGCAAACTAACAAC
3	000016617	fedi000064895	ATAGTGGCACGTATAGGCA	AACACCGAACACGAGAAT
4	000016230	fedi000002177	GGGTCAGAGTCAGTGGTGT	GCTCCTCAGAGTGGTGCA
5	000000055	fedi000012838	ACAGCCCACAGCAGAGCA	CACCAGCAGCCTGAAGAT
6	000037964	fedi000052155	TACTTCATGCCATGCTCC	AACCTCGACGATCTCAAA
7	000003195	fedi000017309	TGCCCACATTATATGCGGAC	GGAGGAGAATGGGATACAGG
8	000003633	fedi000011424	CCATGTTGGTTGTGAGGCAT	TGGGCTTGTGACAGATACG
9	000003916	fedi000049725	GAGCTATGGGACAAACACTG	TCTGACTTTGCGGTTGGATG
10	000001234	fedi000012474	CCGCTAATGAACATCGTG	TCCTCCGTCTGTAATCCC
11	000004497	fedi000051313	CGATTCCCTGATGAACAAGC	CATTCTCAGACAAGGCGACA
12	000005660	fedi000060066	AGAGCAGAGCCAGTGGAGCA	AGTTCAGGGTCGGCGGAGTA
13 *	M24113.1	M24113.1	TGCAAAGCCGGATTCGCTGG	AGTTGGTGACAATACCGTGC

* common carp beta-actin.

Table S4. Specific genotyping primers in gibel carps to estimate the level of tri-allelic polymorphism.

Loci	*Gibel Carp* Transcript	Forward Primer	Reverse Primer
1	000000377	CCCACCCTCAGAATGTTT	CTTCTCCATTTCACTGCGTC
2	000002772	GCTGATTGGCTCTGGTCTGT	TCCCAGGGCTTCTGATGTT
3	000003185	TCTAGGTCTACAGGGAATGG	AGACGGTAGATGAACTCCCT
4	000003195	TGCCCACATTATATGCGGAC	GGAGGAGAATGGGATACAGG
5	000006001	GCGTTGGGAATCAGAAGAG	AATCCGAACACGAGTCCACG
6	000003781	CCTGAGGCAGCATGTGATT	GTACTCCGTCTCATCCGCAT
7	000003916	GAGCTATGGGACAAACACTG	TCTGACTTTGCGGTTGGATG
8	000003932	ACCACAGCCCAGCACATTCT	TCAGCCGTAACGCAACCT
9	000001091	ACCCTCGAGCTGGATTAT	CGCACACGCACATTTAGTCA
10	000001234	CCGCTAATGAACATCGTG	TCCTCCGTCTGTAATCCC
11	000001414	GACTCGCAGCAGTTGGTT	CATTGGGATGGAGGAAGA
12	000004045	TTGAGCACAGAGGCTGGTTC	GGGCTGCTACTGAAGACATACA
13	000004497	CGATTCCCTGATGAACAAGC	CATTCTCAGACAAGGCGACA
14	000005660	AGAGCAGAGCCAGTGGAGCA	AGTTCAGGGTCGGCGGAGTA
15	000006377	AGATGCTTGAACTTGAGAACCCTG	AGGCAAGGCACTATACAACG
16	000006392	ACTGGATGGAGTGGGTGCTT	GGTTTGTGGTGTCAAAGGGT
17	000007745	TAACCGTGCGACCCGTGATA	AATATCGCCTTCAGCCCACA
18	000033229	GTTACAGCGGGAAATCAAGC	CCAGCAGAGCATTCCATACG

Table S5. Purity Ratios (*A260/A280*) of four RNA samples.

Sample	*A260*/*A280*
pooled tissues of triploid gibel carp	2.010
pooled tissues of diploid crucian carp	2.128
gonad of triploid gibel carp	2.039
gonad of diploid crucian carp	2.079

Table S6. RIN values of four RNA samples.

Sample	RIN
pooled tissues of triploid gibel carp	7.8
pooled tissues of diploid crucian carp	7.8
gonad of triploid gibel carp	8
gonad of diploid crucian carp	8

Table S7. Specific RT-qPCR primers of twenty DEGs to validate their expression differences.

Function	Gene Symbol	Forward Primer	Reverse Primer
Immune response	*lect2l*	GGACGACCGCAGAGAAGATA	GCGGTTACTTGAGTTCCCAC
	cbln14	TAACCCTCCCAAGCAAGACA	AGTAGGTGTCCACTGAAGGT
	c5	TGCCCCTTGTTTTCATGTGG	TGGTTCGTGGAGAGCAGATT
	c7	ATGTCCTTGGAGAACAGCCA	TCAGGGTCCTCACAGCTTTT
	vtnb	ACGGAGAAAGAAGACCAGGG	GTCATCCGGGTCATAGCTCA
	tesca	CGCAACTTTTCTGGGACGAA	TAGTCACTGTAGAGCTGCCG
	c8b	CTTTGGCGTTTTCTGCTCCT	GTCCTTCTTCAGACCACCCA
Junction proteins	*cx32.3*	GGCAGTTCCCCATCTCTCAT	TGGGTTAGATAGCTGCCGAG
	sid4	GCTTCTCTCTGCTTTCCTGC	AGTGATGGTCAGGACAGCTC
	itga6b	CTCGTTGGCATGACTGACTG	CAGCTTGTTCGGTTTCTCCC
Transferases	*vtna*	CTTTGCCATTCCTGCTCACA	CTCAGCCCATCGGTCATAGT
	tat	CACACGGATTGGATGGGTTG	GCCATCAGCGTGTTGTGTAA
	cetp	AGTCAAGTCATGCCAGCTCT	CTCCTCCATCTCAGTGGGTC
Binding proteins	*fabp11a*	ATCCCAGGTCTGTTTCTGCA	ACCTGATCGTGTGTGTGGAT
	rbp2b	AGATTGCAGCCCACCTTACT	CCCAGGTCACCAGAGTCTTT
Ubiquitin related proteins	*ube3a*	ACACTCTACATTGGGCGACA	GAAACGGCCTCTGGTTCATC
Transporters	*Tnpo1*	TCACCTCGCAGTCTTCATCA	GCTCTGAATCGACCCCTCAA
	TMEM87A	AGAAGGAGGGTTGCAGTGAA	TTGGCAGGTGGATGATTTGC
	accn2	TCCGTAGCGTGTGAAGATCA	CACTTGGTAGAGGAGAGCGT
Signaling Proteins	*IQGAP1*	TCGAACAGCTTCATCACTGC	GAAATCGAGGATCTGCAGCC
	srp54	TTTGCCAAACCAACAACCCT	GGGCGGAAGAAGATGGTTTT
	Pcsk6	ATCCCACCACCTCTCCTTTG	GACCTGCGTTCCTGAATGTG
	Map4k4	ATATAAGCGACGGAGGTGGG	ATGCAGTGGATGTGGACTCA
	smarcb1a	CCAGTCTGGCCTGTAGTCAA	ACGGTCATCCCAAGCTTTTG
Fish-egg lectin-like precursor	*fel*	ATGTGAGCTTTGACCTGGGA	GTCACTTTGAGGCTTCGGTC

© 2014 by the authors; licensee MDPI, Basel, Switzerland. This article is an open access article distributed under the terms and conditions of the Creative Commons Attribution license (http://creativecommons.org/licenses/by/3.0/).
